# A meta-analysis of hypoxia inducible factor 1-alpha (HIF1A) gene polymorphisms: association with cancers

**DOI:** 10.1186/s40364-015-0054-z

**Published:** 2015-12-29

**Authors:** Md. T Anam, Alokta Ishika, Md. B Hossain, Jesmin

**Affiliations:** Department of Statistics, Biostatistics & Informatics, University of Dhaka, Dhaka, 1000 Bangladesh; Department of Genetic Engineering & Biotechnology, University of Dhaka, Dhaka, 1000 Bangladesh

**Keywords:** HIF1A, Genome wide association studies, Cancer, Meta-analysis

## Abstract

**Background:**

Hypoxia inducible factor 1-alpha (HIF1A) is a transcription factor that plays important role in regulating cascade of reactions. In this study, the effect of rs11549465 (1772 C/T) and rs11549467 (1790 G/A) polymorphisms of HIF1A gene and its association with cancers were investigated through meta-analysis.

**Methods:**

Meta-analysis of genome wide association studies of HIF1A 1772 C/T polymorphism were conducted on 22 case-control studies of sample size 19024 and for 1790 G/A polymorphism 19 case-control studies were included with sample size 10654. Genotype and allelic frequency compared between cases and controls together with further subgroup analyses were carried out by cancer type and ethnicity.

**Results:**

Meta-analysis from this study indicated that HIF1A 1772 C/T polymorphism is significantly associated with overall cancer risk. T allele and genotype TT are significantly associated with increasing overall cancer risk; odds ratios (OR) dominant model [TT + CT vs. CC: OR 1.30, 95 % CI (1.06-1.59), *p*-value: 0.0115], and T allele vs. C allele: OR 1.32, 95 % CI (1.07-1.63), *p*-value: 0.0098. Also, HIF1A 1790 G/A polymorphism, analyses showed that A allele and genotype AA are significantly associated with increasing overall cancer risk; odds ratios (OR) homozygote comparison [AA vs. GG: OR 5.10, 95 % CI (3.12-8.33), *p*-value: <0.0001], heterozygote comparison [GA vs. GG: OR 1.74, 95 % CI (1.20-2.52), *p*-value: 0.0033], dominant model [AA + GA vs. GG: OR 1.82, 95 % CI (1.26-2.62), *p*-value: 0.0014], recessive model [AA vs. GA + GG: OR 3.79, 95 % CI (2.34-6.15), *p*-value: <0.0001] and A allele vs. G allele: OR 1.82, 95 % CI (1.31-2.52), *p*-value: 0.0003.

**Conclusion:**

In detail meta-analysis indicated that both the polymorphisms 1772 C/T and 1790 G/A are significantly associated with overall cancer risk. The subgroup analyses showed that lung cancer is significantly associated with both polymorphisms. Although the 1772 C/T polymorphism is significantly associated with decreasing risk of renal cell carcinoma but the 1790 G/A polymorphism has shown to significantly increase the cancer risk in both Caucasian and Asian population. Thus, HIF1A could be a useful prognostic marker for cancers early predisposition.

**Electronic supplementary material:**

The online version of this article (doi:10.1186/s40364-015-0054-z) contains supplementary material, which is available to authorized users.

## Background

Cancer is the second leading cause of morbidity and mortality worldwide [1]. One major feature of cancer is uncontrolled cell proliferation, which can then invade adjacent parts of the body and spread to other organs, the latter process is referred as metastases, which are the major cause of death from cancer [2]. The most common causes of cancer deaths are due to cancers of the: lung (1.59 million deaths), liver (745,000 deaths), stomach (723,000 deaths), colorectal (694,000 deaths), breast (521,000 deaths) and esophageal (400,000 deaths) [1, 2]. Alongside, metabolic alterations and tumor hypoxia have consistently been identified as classical features with aggressive malignancy [3, 4]. Hypoxia regulates tumor cell phenotype mainly by altering genes that are sensitive to oxygen pressure [5]. However, the exact mechanism of carcinogenesis is yet to be elucidated. In recent years, an increasing number of studies have focused on understanding the relationship between genetic factors and cancer risk [3, 4]. Through the years, it has become well accepted that single nucleotide polymorphisms (SNPs) are the most common and effective type of genetic variations studied in association with disease susceptibility and are the markers of many complex diseases [6].

Hypoxia inducible factor 1α (HIF1A), is a transcription factor that has major impacts in the process of development and progression of cancers [7]. HIF1A regulates the expression of over 100 genes that control the major cellular functions including apoptosis, cell proliferation, glucose metabolism, erythropoiesis, iron metabolism and angiogenesis. It is a master regulator of oxygen homeostasis [7]. In the scientific community, HIF1A has been a research focus and a number of SNPs within HIF1A gene have been identified in association with cancers, with the most widely studied polymorphisms are C1772T (rs11549465) and G1790A (rs11549467) polymorphisms [8–38]. These two SNPs are located within the same domain (ODD/ pVHL) in exon 12 of the HIF1A gene [8, 9]. Recently a meta-analysis has revealed that C1772T is not in substantial linkage disequilibrium (LD) with G1790A [38]. A number of studies have suggested that these two nonsynonymous mutations might alter the transcriptional activity of HIF1A gene by causing structural changes with varied stability, which in turn, might influence the downstream target genes expression and regulation [8, 9, 38]. In the recent years, a good number of studies have investigated the impact of HIF1A polymorphisms on cancer risk in different populations; however reported results varied across studies and remain inconclusive [10–38]. In this study, the effect of rs11549465 (1772 C/T) and rs11549467 (1790 G/A) polymorphisms of HIF1A gene and its association with cancers were investigated systematically through meta-analysis.

## Methods

### Search study and study selection

The PubMed, PubMed Central and Google Scholar databases were searched systematically to retrieve compatible and pertinent peer reviewed publications of empirical studies. Published articles of last 15 years (ended on December 2014), in English language were only considered for this study. The search terms included were (1) HIF1A, (2) GWAS, (3) SNPs, (4) polymorphisms, (5) C1772T/ P582S, (6) A1790G/ A588T, (7) case-control study, and (8) cancer.

### Eligibility criteria

**Fig. 1:**
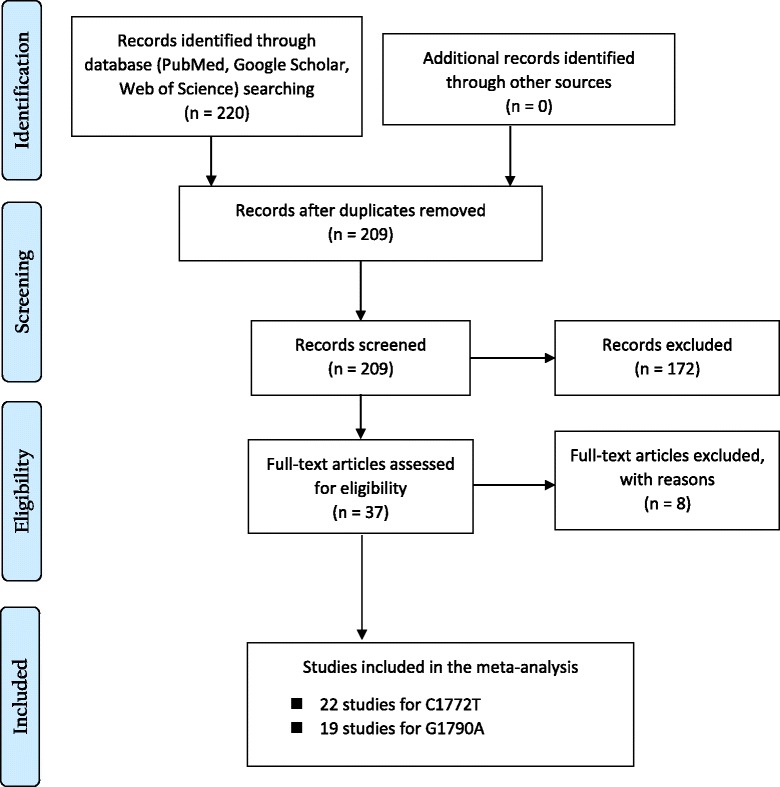
Flow diagram of study selection for HIF1A 1772 C/T and 1790 G/A polymorphisms; where “n” in the boxes is the number of corresponding studies

Two authors independently investigated titles and abstracts of all the articles. Irrelevant and incompatible studies were excluded primarily. For final review, criteria’s for further study elimination were: if (1) the study population was not defined completely; (2) it is not a case-control study; (3) not a genome wide association study; (4) incomplete information of allele frequency; and (5) the year of study conducted was not specified. Also, reviews, editorials, meta-analysis and non-human researches were excluded. Only case-control studies, genome wide association study (GWAS) and human researches were considered for the final review. Further, the references of the selected studies were screened carefully for incorporation of additional relevant studies. Only English language articles were considered for this study. Discrepancies and difficulties were discussed with corresponding authors where necessary. Following information were extracted from each study: (1) authors name, (2) year of study, (3) ethnicity of the study subjects, (4) cancer type and (5) allelic frequency (Fig. 1).

### Meta-analysis

For HIF1A 1772 C/T polymorphism 22 case-control studies were included of sample size 19024 and for 1790 G/A polymorphism 19 case-control studies were included with sample size 10654. The meta-analysis was prepared in accordance with PRISMA statement [39].

### Statistical analysis

Meta-analysis of genome wide association studies (GWAS) of HIF1A were conducted for two polymorphisms, 1772 C/T and 1790 G/A using odds ratios (ORs). A slightly amended estimator of OR was used to avoid the computation of reciprocal of zeros among observed values in the calculation of the original OR [40]. Pooled ORs with 95 % CIs were calculated using random effects model (REM) incorporating the inverse variance weighted method [41]. Heterogeneity among studies was assessed using the Q statistic [42] and quantified using I^2 index [43]. Subgroup analyses were carried out by cancer type and ethnicity. The Hardy Weinberg Equilibrium (HWE) test was performed for the controls of each study. The studies with control not in HWE were supervised for sensitivity analysis. Publication bias was assessed visually by conventionally constructed funnel plot where the inverse of the standard error (1/se) of the effect estimates were plotted against the logarithm transformation of Odds Ratios [log(OR)] [44]. Furthermore, Egger’s test was performed to provide quantitative evidence of publication bias [45]. “Gap: Genetic analysis package” was used to perform the Hardy Weinberg Equilibrium (HWE) test [46, 47]. All analyses were conducted using “meta” package in R environment [46].

### Summary measures

Odds Ratios (OR) with a 95 % confidence interval (CI) were calculated to evaluate the genotype contrasts. The genotype contrasts for the HIF1A 1772 C/T polymorphisms were: homozygote comparison [TT versus CC], heterozygote comparison [CT versus CC], and dominant model [TT + CT versus CC], recessive model [TT versus CT + CC] and T allele versus C allele. For HIF1A 1772 C/T polymorphism, three studies were found with genotype information of CC and CT + TT. These three studies were included only to evaluate genotype contrast of dominant model [TT + CT vs. CC]. The genotype contrasts for the HIF1A 1790 G/A polymorphism were: homozygote comparison [AA versus GG], heterozygote comparison [GA versus GG] and dominant model [AA + GA versus GG], recessive model [AA versus GA + GG] and [G versus A allele].

## Results and discussion

### Study characteristics

In the meta-analysis of the HIF1A 1772 C/T polymorphism, ten different types of cancers consisted of 22 studies with 8149 cancer cases and 10,875 controls were included. The types of cancer included in these studies were prostate cancer, colorectal cancer, renal cell carcinoma, breast cancer, lung cancer, oral squamous cell carcinoma (OSCC), head-neck cancer, cervical cancer, bladder carcinoma and pancreatic cancer. For the following cancer types: head-neck, cervical, bladder and pancreatic only one study of each were found for the final review. So, these cancer types with single studies were incorporated in subgroup analysis as Other Cancers (Table 1).

**Table 1:** Characteristic of eligible studies included in meta-analysis of HIF1A 1772 C/T polymorphism

Study	Year	Country	Ethnicity	Cancer	Case/Control	HWE
Clifford et al. [8]	2001	UK	Caucasian	Renal cell carcinoma	35/143	0.018 (N)
Tanimoto et al. [9]	2003	Japanese	Asian	Head-neck cancer	55/110	0.545 (Y)
Ollerenshawa et al. [10]	2004	European	Caucasian	Renal cell carcinoma	160/162	<0.001 (N)
Chau et al. [11]	2005	USA	Mixed	Prostate cancer	196/196	<0.001 (N)
Franse et al. [12]	2006	Swedish	Caucasian	Colorectal cancer	198/258	0.916 (Y)
Konac et al. [13]	2007	Turkish	Caucasian	Cervical cancer	32/107	0.229 (Y)
Li et al. [14]	2007	American	Mixed	Prostate cancer	1041/1234	0.159 (Y)
Lee et al. [15]	2008	Korean	Asian	Breast cancer	1332/1369	0.250 (Y)
Kim et al. [16]	2008	Korean	Asian	Breast cancer	90/102	0.641 (Y)
Nadaoka et al.^a^ [17]	2008	Japanese	Asian	Transitional cell carcinoma of bladder	219/461	
Jacobs et al. [18]	2008	American	Mixed	Prostate cancer	1420/1450	0.041 (N)
Foley et al. [19]	2009	Ireland	Caucasian	Prostate cancer	95/188	0.623 (Y)
Morris et al. [20]	2009	Polish	Caucasian	Renal cell carcinoma	332/313	0.083 (Y)
Chen et al. [21]	2009	Taiwanese	Asian	Oral squamous cell carcinoma (OSCC)	174/347	0.722 (Y)
Shieh et al. [22]	2010	Taiwan	Asian	Oral squamous cell carcinoma (OSCC)	305/96	0.710 (Y)
Knechtel et al.^a^ [23]	2010	Austria	Caucasian	Colorectal cancer	368/2156	
Kang et al.^a^ [24]	2011	Korean	Asian	Colorectal cancer	50/50	
Putra et al. [25]	2011	Japanese	Asian	Lung cancer	83/110	0.545 (Y)
Wang et al. [26]	2011	Chinese	Asian	Pancreatic cancer	263/271	0.352 (Y)
Kuo et al. [27]	2012	Taiwanese	Asian	Lung cancer	285/300	0.132 (Y)
Li et al. [28]	2012	China	Asian	Prostate cancer	662/716	0.267 (Y)
Fraga et al. [29]	2014	Portuguese	Caucasian	Prostate cancer	754/736	0.400 (Y)

^a^Frequency of genotypes “CT + TT”. *HWE* Hardy-Weinberg Equilibrium

For the meta-analysis of HIF1A 1790 G/A polymorphism, 19 studies with eleven different cancer types consisted of 4681 cancer cases and 5973 controls were included. The cancer types associated with this polymorphism were: renal cancer, prostate cancer, breast cancer, lung cancer, oral squamous cell carcinoma (OSCC), head-neck cancer, gastric cancer, hepatocellular carcinoma, lymph node metastasis, pancreatic cancer and colorectal cancer. For final review, only one study of each of the following cancer types was found: head-neck cancer, gastric cancer, hepatocellular carcinoma, lymph node metastasis, pancreatic cancer and colorectal cancer. These cancer types with single studies were incorporated in subgroup analysis as Other Cancers (Table 2).

**Table 2:** Characteristic of eligible studies included in meta-analysis of HIF1A 1790G/A polymorphism

Study	Year	Country	Ethnicity	Cancer	Case/Control	HWE
Clifford et al. [8]	2001	Caucasian	Caucasian	Renal cancer	48/144	0.866(Y)
Tanimoto et al. [9]	2003	Japan	Asian	Head neck squeamish cell carcinoma	55/110	0.655(Y)
Ollerenshaw et al. [10]	2004	Caucasian	Caucasian	Renal cancer	146/288	<0.001(N)
Fransen et al. [12]	2006	Sweden	Caucasian	Colorectal cancer	198/256	0.775(Y)
Orr-Urtreger et al. [30]	2007	Israel	Caucasian	Prostate cancer	200/300	0.954(Y)
Li et al. [14]	2007	USA	Mixed	Prostate cancer	1066/1264	0.810(Y)
Apaydin et al. [31]	2008	Turkey	Caucasian	Breast cancer	102/102	0.840(Y)
Kim et al. [16]	2008	Korea	Asian	Breast cancer	90/102	0.06(Y)
Muñoz et al. [32]	2009	Spain	Caucasian	Oral squamous cell carcinoma	64/139	0.693(Y)
Chen et al. [21]	2009	Taiwanese	Asian	Oral squamous cell carcinoma	174/347	0.701(Y)
Morris et al. [20]	2009	polish	Caucasian	Renal cancer	325/309	0.662(Y)
Li K et al. [33]	2009	Tibetan	Asian	Gastric cancer	87/106	0.764(Y)
Hsiao et al. [34]	2010	Taiwan	Asian	Hepatocellular carcinoma	102/347	0.701(Y)
Putra et al. [25]	2011	Japan	Asian	Lung cancer	83/110	0.655(Y)
Wang et al. [26]	2011	Japan	Asian	Pancreatic cancer	263/271	0.486(Y)
Kuo et al. [27]	2012	China	Asian	Lung cancer	285/300	0.154(Y)
Li et al. [28]	2012	China	Asian	Prostate cancer	662/716	0.554(Y)
Mera-Mene et al. [35]	2012	Spain	Caucasian	Lymph node metastasis	111/139	0.693(Y)
Qin et al. [36]	2012	Asian	Asian	Renal cancer	620/623	0.411(Y)

*HWE* Hardy-Weinberg Equilibrium

### Association of the HIF1A 1772 C/T polymorphism with cancer risk

**Fig. 2:**
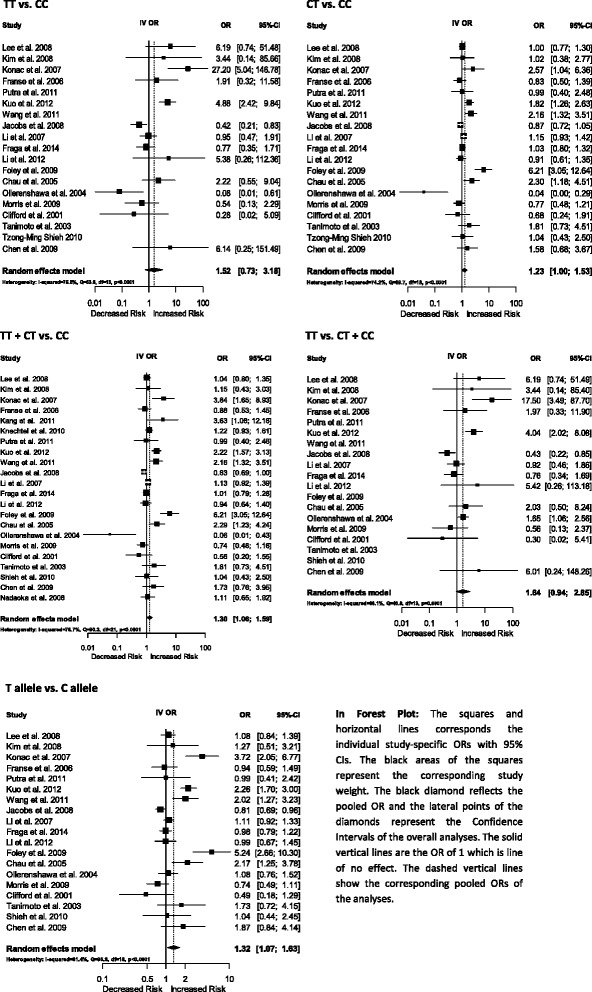
Forest plot of HIF1A polymorphism 1772 C/T for overall cancer

The pooled ORs for overall cancer suggested that the HIF1A 1772 C/T polymorphism was significantly associated with increasing cancer risk for the dominant model [TT + CT vs. CC: OR 1.30, 95 % CI (1.06-1.59), *p*-value: 0.0115] and [T vs. C allele: OR 1.32, 95 % CI (1.07-1.63), *p*-value: 0.0098] (Fig. 2).

#### Subgroup analyses performed by cancer type

The subgroup analyses of prostate cancer, colorectal cancer, breast cancer and oral squamous-cell carcinoma suggested no significant association of the HIF1A 1772 C/T polymorphism. However, the subgroup analyses of renal cell carcinoma suggested that the HIF1A 1772 C/T polymorphism is significantly associated with lowering renal cell carcinoma risk in homozygote comparison [TT vs. CC: OR 0.27, 95 % CI (0.08-0.90), *p*-value:0.0335]. Interestingly, the results of subgroup analyses of lung cancer suggested that the HIF1A 1772 C/T polymorphism is highly associated with increasing lung cancer risk in homozygote comparison [TT vs. CC: OR 4.88, 95 % CI (2.42-9.84), *p*-value: <0.0001], recessive model [TT vs. CT + CC: OR 4.04, 95 % CI (2.02-8.08), *p*-value:<0.0001]. The subgroup analyses of Other Cancers suggested that the HIF1A 1772 C/T polymorphism is highly associated with increasing Other Cancer risk in homozygote comparison [TT vs. CC: OR 27.20, 95 % CI (5.04-146.78), *p*-value: 0.0001], heterozygote comparison [CT vs. CC: OR 2.16, 95 % CI (1.46-3.18), *p*-value: 0.0056], dominant model [TT + CT vs. CC: OR 1.92, 95 % CI (1.17-3.14), *p*-value: 0.0093], recessive model [TT vs. CT + CC: OR 17.5, 95 % CI (3.49-87.70), *p*-value: 0.0005] and [T vs. C allele: OR 2.42, 95 % CI (1.55-3.77), *p*-value: <0.0001] (Table 3).

**Table 3:** Meta-analysis of the HIF1A 1772 C/T polymorphism association with cancer

			TT vs. CC		CT vs. CC		TT + CT vs. CC		TT vs. CT + CC		T vs. C	
	Study number	Sample size	OR (95 % CI)	*p* value	OR (95 % CI)	*p* value	OR (95 % CI)	*p* value	OR (95 % CI)	*p* value	OR (95 % CI)	*p* value
Overall cancer	22	19024	1.52 [0.73–3.18]	0.2648	1.23 [1.00–1.53]	0.0536	1.30 [1.06–1.59]	0.0115	1.64 [0.94–2.85]	0.0832	1.32 [1.07–1.63]	0.0098
Prostate cancer	6	8688	0.84 [0.47–1.49]	0.5449	1.34 [0.95–1.87]	0.0913	1.33 [0.95–1.87]	0.0982	0.81 [0.47–1.40]	0.4535	1.29 [0.94–1.76]	0.1178
Colorectal cancer	3	3080	1.91 [0.32–11.58]	0.4801	0.83 [0.50–1.39]	0.4817	1.24 [0.77–2.01]	0.3756	1.97 [0.33–11.90]	0.4603	0.94 [0.59–1.49]	0.7833
Renal cancer	3	1145	0.27 [0.08–0.90]	0.0335	0.40 [0.12–1.34]	0.1369	0.43 [0.15–1.20]	0.1082	1.08 [0.44–2.64]	0.8703	0.84 [0.58–1.22]	0.3548
Breast cancer	2	2893	5.18 [0.88–30.38]	0.0683	1.00 [0.77–1.29]	0.9964	1.05 [0.81–1.35]	0.7221	5.18 [0.88–30.36]	0.0684	1.09 [0.86–1.39]	0.4701
Lung cancer	2	778	4.88 [2.42–9.84]	< 0.0001	1.56 [0.94–2.61]	0.088	1.67 [0.79–3.54]	0.1832	4.04 [2.02–8.08]	< 0.0001	1.68 [0.77–3.64]	0.1908
OSCC	2	922	6.14 [0.25–151.49]	0.2673	1.29 [0.70–2.37]	0.4142	1.36 [0.75–2.49]	0.3127	6.01 [0.24–148.26]	0.2729	1.43 [0.79–2.56]	0.2348
Other cancers	4	1518	27.20 [5.04–146.78]	0.0001	2.16 [1.46–3.18]	0.0056	1.92 [1.17–3.14]	0.0093	17.5 [3.49 – 87.70]	0.0005	2.42 [1.55–3.77]	< 0.0001
Ethnicity												
Caucasian	8	6037	0.97 [0.24–3.93]	0.9654	1.09 [0.60–2.00]	0.7751	1.19 [0.75–1.89]	0.4528	1.48 [0.65–3.39]	0.352	1.31 [0.84–2.06]	0.237
Asian	11	7450	4.98 [2.66–9.31]	< 0.0001	1.30 [1.01–1.69	0.0455	1.41 [1.08–1.84]	0.0109	4.28 [2.31–7.95]	< 0.0001	1.43 [1.07–1.90]	0.0156
Mixed	3	5537	0.82 [0.36–1.87]	0.6408	1.16 [1.00–1.65]	0.4178	1.16 [0.79–1.70]	0.4526	0.79 [0.37–1.71]	0.5544	1.14 [0.78–1.67]	0.505

#### Subgroup analyses by ethnicity group

The analyses data for the HIF1A 1772 C/T polymorphism suggested that there was no significant effect on the Caucasian population. However, the subgroup analyses of the Asian population suggested that the HIF1A 1772 C/T polymorphism was significantly associated with increasing cancer risk in homozygote comparison [TT vs. CC: OR 4.98, 95 % CI (2.66-9.31), *p*-value: <0.0001], heterozygote comparison [CT vs. CC: OR 1.30, 95 % CI (1.01-1.69), *p*-value: 0.0455], dominant model [TT + CT vs. CC: OR 1.41, 95 % CI (1.08-1.84), *p*-value: 0.0109], recessive model [TT vs. CT + CC: OR 4.28, 95 % CI (2.31-7.95), *p*-value:<0.0001] and [T vs. C allele: OR 1.43, 95 % CI (1.07-1.90), *p*-value: 0.0156] (Table 3). The subgroup analyses of mixed ethnic groups suggested that there were no significant association between HIF1A 1772 C/T polymorphism and cancer risk (Table 3).

### Sources of heterogeneity

There were significant heterogeneity observed in the analyses of HIF1A 1772 C/T polymorphism for overall cancer heterozygote comparison [CT vs. CC: Q = 69.67, d.f = 18, *p*-value 0.0001, I^2 = 74.2 % (59.5 %-83.5 %)], dominant model [TT + CT vs. CC: Q = 90.25, d.f = 21, *p* <0.0001, I^2 = 76.7 % (65.1 %-84.5 %)], and [T vs. C allele: Q = 96.87, d.f = 18, *p* <0.0001, I^2 = 81.4 % (71.9 %-87.7 %). To detect the sources of heterogeneity subgroup analyses by cancer type and ethnicity group were performed. In the subgroup analyses by cancer type heterogeneity was significantly reduced. The results suggested that the studies in prostate cancer, renal cell carcinoma, lung cancer, Caucasian ethnicity and Asian ethnicity were the main sources of heterogeneity (Additional file 1).

### Association of the HIF1A 1790 G/A polymorphism with cancer risk

**Fig. 3:**
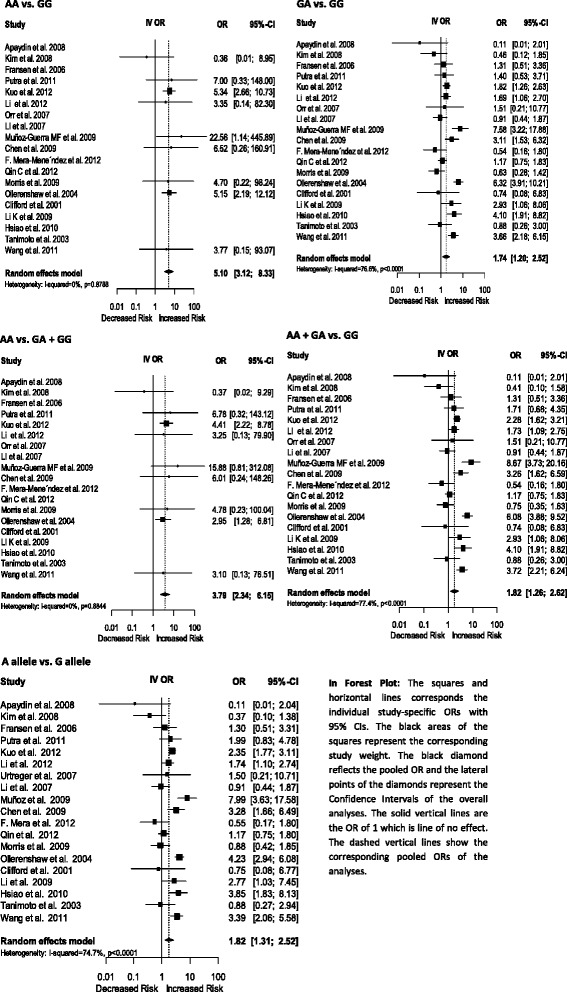
Forest plot of the HIF1A polymorphism 1790 G/A for overall cancer

The pooled ORs for overall cancer suggested that the HIF1A 1790 G/A polymorphism was significantly associated with increasing cancer risk for homozygote comparison [AA vs. GG: OR 5.10, 95 % CI (3.12-8.33), *p*-value: <0.0001, heterozygote comparison [GA vs. GG: OR 1.74, 95 % CI (1.20-2.52), *p*-value: 0.0033, dominant model [AA + GA vs. GG: OR 1.82, 95 % CI (1.26-2.62), *p*-value: 0.0014], recessive model [AA vs. GA + GG: OR 3.79, 95 % CI (2.34-6.15), *p*-value: <0.0001] and [A vs. G allele: OR 1.82, 95 % CI (1.31-2.52), *p*-value: 0.0003] (Fig. 3).

#### Subgroup analyses by cancer type

The analyzed data of prostate cancer suggested no significant association with the HIF1A 1790 G/A polymorphism. The subgroup analyses of renal cancer suggested that the HIF1A 1790 G/A polymorphism was significantly associated with increasing cancer risk for homozygote comparison [AA vs. GG: OR 5.11, 95 % CI (2.24-11.66), *p*-value: 0.0001], recessive model [AA vs. GA + GG: OR 3.05, 95 % CI (1.36-6.84), *p*-value: 0.0068] whereas the subgroup analyses of breast cancer showed that the HIF1A 1790 G/A polymorphism was significantly associated with decreasing cancer risk for [A vs. G allele: OR 0.30, 95 % CI (0.09-1.00), *p*-value: 0.0495]. The subgroup analyses of lung cancer suggested that the HIF1A 1790 G/A polymorphism was significantly associated with increasing cancer risk for homozygote comparison [AA vs. GG: OR 5.41, 95 % CI (2.74-10.69), *p*-value: <0.0001], heterozygote comparison [GA vs. GG: OR 1.76, 95 % CI (1.25-2.49), *p*-value: 0.0013], dominant model [AA + GA vs. GG: OR 2.20, 95 % CI (1.60-3.03), *p*-value:<0.0001], recessive model [AA vs. GA + GG: OR 4.51, 95 % CI (2.31-8.81), *p*-value:<0.0001] and [A vs. G allele: OR 2.31, 95 % CI (1.77-3.02), *p*-value: <0.0001]. Also, the subgroup analyses of oral squamous cell carcinoma (OSCC) suggested that the HIF1A 1790 G/A polymorphism was significantly associated with increasing cancer risk for homozygote comparison [AA vs. GG: OR 12.68, 95 % CI (1.43-112.64), *p*-value: 0.0227], heterozygote comparison [GA vs. GG: OR 4.69, 95 % CI (1.96-11.21), *p*-value: 0.0005], dominant model [AA + GA vs. GG: OR 5.17, 95 % CI (1.99-13.43), *p*-value: 0.0008], recessive model [AA vs. GA + GG: OR 10.12, 95 % CI (1.14-89.72), *p*-value: 0.0376] and [A vs. G allele: OR 5.00, 95 % CI (2.10-11.97), *p*-value: 0.0003] (Table 4). The subgroup analyses of Other Cancers suggested that the HIF1A 1790 G/A polymorphism is highly associated with increasing Other Cancer risk heterozygote comparison [GA vs. GG: OR 1.96, 95 % CI (1.05-3.65), *p*-value: 0.0336], dominant model [AA + GA vs. GG: OR 1.96, 95 % CI (1.05-3.67), *p*-value: 0.0341], and [A vs. G allele: OR 1.91, 95 % CI (1.06-3.44), *p*-value: 0.0306] (Table 4).

**Table 4:** Meta-analysis of the HIF1A 1790 G/A polymorphism association with cancer

			AA vs. GG		GA vs. GG		AA vs. GA + GG		AA + GA vs. GG		A vs. G	
	Study number	Sample size	OR (95 % CI)	*p* value	OR (95 % CI)	*p* value	OR (95 % CI)	*p* value	OR (95 % CI)	*p* value	OR (95 % CI)	*p* value
Overall	19	10654	5.10 [3.12–8.33]	< 0.0001	1.74 [1.20–2.52]	0.0033	3.79 [2.34–6.15]	< 0.0001	1.82 [1.26–2.62]	0.0014	1.82 [1.31–2.52]	0.0003
Renal cancer	4	2503	5.11 [2.24–11.66]	0.0001	1.51 [0.45–5.05]	0.5038	3.05 [1.36–6.84]	0.0068	1.58 [0.49–5.03]	0.442	1.53 [0.60–3.92]	0.3747
Prostate cancer	3	4208	3.35 [0.14–82.30]	0.4597	1.41 [0.96–2.08]	0.0822	3.25 [0.13–79.90]	0.4707	1.41 [0.93–2.15]	0.1043	1.42 [0.93–2.17]	0.1093
Breast cancer	2	396	0.36 [0.01–8.95]	0.5332	0.35 [0.10–1.24]	0.1045	0.37 [0.02–9.29]	0.5484	0.32 [0.09–1.10]	0.0702	0.30 [0.09–1.00]	0.0495
Lung cancer	2	778	5.41 [2.74–10.69]	< 0.0001	1.76 [1.25–2.49]	0.0013	4.51 [2.31–8.81]	< 0.0001	2.20 [1.60–3.03]	< 0.0001	2.31 [1.77–3.02]	< 0.0001
OSCC	2	724	12.68 [1.43–112.64]	0.0227	4.69 [1.96–11.21]	0.0005	10.12 [1.14–89.72]	0.0376	5.17 [1.99–13.43]	0.0008	5.00 [2.10–11.97]	0.0003
Other cancers	6	2045	3.77 [0.15–93.07]	0.4171	1.96 [1.05–3.65]	0.0336	3.10 [0.13–76.51]	0.4887	1.96 [1.05–3.67]	0.0341	1.91 [1.06–3.44]	0.0306
Ethnicity												
Caucasian	8	2666	5.68 [2.57–12.58]	< 0.0001	1.43 [0.54–3.74]	0.4691	3.42 [1.57–7.45]	0.002	1.50 [0.58–3.85]	0.3987	1.52 [0.68–3.42]	0.3103
Asian	10	4914	4.76 [2.55–8.91]	< 0.0001	1.94 [1.38–2.72]	0.0001	4.05 [2.1 –7.51]	< 0.0001	2.04 [1.44–2.87]	< 0.0001	2.03 [1.46–2.81]	< 0.0001

#### Subgroup analyses by ethnicity group

For Caucasian population, the analyzed data suggested that the HIF1A 1790 G/A polymorphism was highly associated with increasing cancer risk for homozygote comparison [AA vs. GG: OR 5.68, 95 % CI (2.57-12.58), *p*-value: <0.0001], recessive model [AA vs. GA + GG: OR 3.42, 95 % CI (1.57-7.45), *p*-value: 0.002]. For the Asian population, the subgroup analyses of ethnicity group suggested that the HIF1A 1790 G/A polymorphism was highly associated with increasing cancer risk for homozygote comparison [AA vs. GG: OR 4.76, 95 % CI (2.55-8.91), *p*-value: <0.0001], heterozygote comparison [GA vs. GG: OR 1.94, 95 % CI (1.38-2.72), *p*-value: 0.0001], dominant model [AA + GA vs. GG: OR 2.04, 95 % CI (1.44-2.87), *p*-value: <0.0001], recessive model [AA vs. GA + GG: OR 4.05, 95 % CI (2.18-7.51), *p*-value: <0.0001] and [A vs. G allele: OR 2.03, 95 % CI (1.46-2.81), *p*-value: <0.0001] (Table 4).

### Sources of heterogeneity

There were significant heterogeneity observed in the analyses of HIF1A 1790G/A polymorphism for overall cancer heterozygote comparison [GA vs. GG: Q = 77.05, d.f = 18, *p*-value: <0.0001, I^2 = 76.6 % (63.8 %-84.9 %), dominant model [AA + GA vs. GG: Q = 79.66, d.f = 18, *p*-value: <0.0001, I^2 = 77.4 % (65.1 %-85.4 %)], and [A vs. G allele: Q = 71.09, d.f = 18, *p*-value: <0.0001, I^2 = 74.7 % (60.4 %-83.8 %)]. To detect the sources of heterogeneity subgroup analyses by cancer type and ethnicity group were performed. The results suggested that the studies in renal cell carcinoma, oral squamous cell carcinoma (OSCC), Caucasian ethnicity and Asian ethnicity were the main sources of heterogeneity (Additional file 2).

### Publication bias

**Fig. 4:**
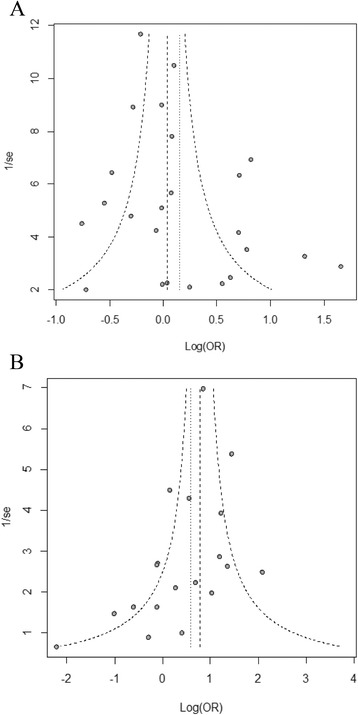
Funnel plot of HIF1A polymorphism (**a**) 1772 C/T for T allele vs. C allele and (**b**) 1790 G/A for A allele vs. G allele; showing visual evidence of publication bias

To investigate the evidence of publication bias of the HIF1A 1772 C/T polymorphism for T versus C allele and HIF1A 1790 G/A polymorphism for G versus A allele funnel plot were used. The conventionally constructed funnel plot (log odds ratio [log(OR] vs 1/standard error, 1/se) of HIF1A polymorphism 1772 C/T for T vs. C allele suggested that there was evidence of publication bias (Fig. 4). Also the funnel plot of HIF1A polymorphism 1790 G/A for A vs. G allele suggested that there was evidence of publication bias (Fig. 4). However, the Egger’s linear regression analyses suggested no evidence of significant publication bias in [T vs C allele: t = 1.83, d.f = 17, *p*-value 0.0847] for HIF1A 1772 C/T polymorphism. Also, for HIF1A 1790 G/A polymorphism results showed no significant evidence of publication bias in [A vs G allele: t = -1.87, d.f = 17, *p*-value 0.0787] (Additional file 3).

### Sensitivity analysis

Studies which were not in HWE were excluded to evaluate the stability of the acquired results. The statistical significance of the results was not shifted after omitting the studies which were not in HWE which confirmed the obtained results of the meta-analysis were stable and robust.

## Conclusion

Results generated from this meta-analysis indicated that both 1772 C/T and 1790 G/A polymorphisms are significantly associated with increasing overall cancer risk. The subgroup analyses by cancer type showed that both 1772 C/T and 1790 G/A polymorphisms have significant association with lung cancer, whereas these two polymorphisms showed no significant association with prostate cancer. In oral squamous cell carcinoma (OSCC) subgroup analyses data showed that only 1790 G/A polymorphism has significant association whereas the HIF1A 1772 C/T polymorphism showed no significant association. However, the 1772 C/T polymorphism has indicated significantly decreased risk in renal cell carcinoma. Also, 1790 G/A polymorphism has increased the cancer risk significantly in both Caucasian and Asian ethnicity. Taken together all analyzed data, HIF1A could be a prognostic marker useful for early detection and diagnosis for cancers. In future, further experimental validations would be necessary to confirm the results.

## Additional files

Additional file 1:
**Heterogeneity analysis of HIF1A 1772 C/T.** (XLSX 14 kb) Additional file 2:
**Heterogeneity analysis of A polymorphism/A.** (XLSX 12 kb) Additional file 3:
**Egger's linear regression analyses of HIF1A 1772 C/T and HIF1A 1790 G/A.** (DOCX 17 kb)

## Abbreviations

GWAS: Genome wide association studies
SNP: Single nucleotide polymorphism
REM: Random effects model
CI: Confidence interval
SE: Standard error
Log: Logarithm
HWE: Hardy-Weinberg Equilibrium
HIF1: Hypoxia- inducible factor -1
HIF1A: Hypoxia- inducible factor -1α
OR: Odds ratio
OSCC: Oral squamous cell carcinoma.
