# Supplementary material for: A meta-analysis of hypoxia inducible factor 1-alpha (HIF1A) gene polymorphisms: association with cancers
Source: Biomark Res. 2015 Dec 29;3:29. doi: 10.1186/s40364-015-0054-z (PMC4693423; doi:10.1186/s40364-015-0054-z)
Supplement: Additional file 3: — Egger's linear regression analyses of HIF1A 1772 C/T and HIF1A 1790 G/A. (DOCX 17 kb) [file 40364_2015_54_MOESM3_ESM.docx]

Hypothesis testing by p value

H_0_ : Null Hypothesis (Symmetry in the funnel Plot)

H_1_: Alternative Hypothesis (Asymmetry in the funnel Plot)

If p-value ≤ 0.05 we Reject Null Hypothesis (H_0_)

If p-value > 0.05 we Accept Null Hypothesis (H_0_) and Reject Alternative Hypothesis (H_1_ )

**T vs. C: (C1772T)**

**Linear regression test of funnel plot asymmetry**

data: T vs C_meta

t = 1.8312, df = 17, p-value = 0.08466

alternative hypothesis: asymmetry in funnel plot

sample estimates:

bias se.bias slope

1.7981472 0.9819555 -0.1518358

**A vs. G :(G1790A)**

**Linear regression test of funnel plot asymmetry**

data: AvsG_meta

t = -1.8705, df = 17, p-value = 0.07873

alternative hypothesis: asymmetry in funnel plot

sample estimates:

bias se.bias slope

-1.5944613 0.8524186 1.2195174
